# Energy‐Supporting Enzyme‐Mimic Nanoscaffold Facilitates Tendon Regeneration Based on a Mitochondrial Protection and Microenvironment Remodeling Strategy

**DOI:** 10.1002/advs.202202542

**Published:** 2022-08-24

**Authors:** Shikun Wang, Zhixiao Yao, Xinyu Zhang, Juehong Li, Chen Huang, Yuanming Ouyang, Yun Qian, Cunyi Fan

**Affiliations:** ^1^ Department of Orthopaedics Shanghai Jiao Tong University Affiliated Sixth People's Hospital Shanghai 200233 China; ^2^ Shanghai Engineering Research Center for Orthopaedic Material Innovation and Tissue Regeneration Shanghai 200233 China; ^3^ Youth Science and Technology Innovation Studio Shanghai Jiao Tong University School of Medicine Shanghai 200233 China; ^4^ Engineering Research Center of Technical Textiles Ministry of Education College of Textiles Donghua University Shanghai 201620 China

**Keywords:** ceria nanozyme, mitochondrial dysfunction, nanoscaffold, oxidative stress, tendon regeneration

## Abstract

Tendon injury is a tricky and prevalent motor system disease, leading to compromised daily activity and disability. Insufficient regenerative capability and dysregulation of immune microenvironment are the leading causes of functional loss. First, this work identifies persistent oxidative stress and mitochondrial impairment in the regional tendon tissues postinjury. Therefore, a smart scaffold incorporating the enzyme mimicry nanoparticle‐ceria nanozyme (CeNPs) into the nanofiber bundle scaffold (NBS@CeO) with porous, anisotropic, and enhanced mechanical properties is designed to innovatively explore a targeted energy‐supporting repair strategy by rescuing mitochondrial function and remodeling the microenvironment favoring endogenous regeneration. The integrated CeNPs scavenge excessive reactive oxygen species (ROS), stabilize the mitochondria membrane potential (ΔΨm), and ATP synthesis of tendon‐derived stem cells (TDSCs) under oxidative stress. In a rat Achilles tendon defect model, NBS@CeO reduces oxidative damage and accelerates structural regeneration of collagen fibers, manifesting as recovering mechanical properties and motor function. Furthermore, NBS@CeO mediates the rebalance of endogenous regenerative signaling and dysregulated immune microenvironment by alleviating senescence and apoptosis of TDSCs, downregulating the secretion of senescence‐associated secretory phenotype (SASP), and inducing macrophage M2 polarization. This innovative strategy highlights the role of NBS@CeO in tendon repair and thus provides a potential therapeutic approach for promoting tendon regeneration.

## Graphical abstract

A smart scaffold incorporating the ceria nanozyme into the nanofiber bundle scaffold (NBS@CeO) with porous, anisotropic, and enhanced mechanical properties is designed to explore a targeted energy‐supporting repair strategy by rescuing mitochondrial function and remodeling microenvironment favoring endogenous regeneration. This innovative strategy highlights the role of NBS@CeO in tendon repair and provides a promising therapeutic approach for promoting tendon regeneration.

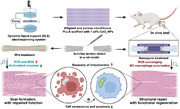

## 1 Introduction

Tendon is the bridge of mechanical transmission between bone and muscle to enable the conversion between mobility and stability. However, the high tensile burdens on the tendon make itself prone to rupture after trauma, which leads to pain, decreased motor capacity, and disability.^[^ ^1^ ^]^ More than 32 million tendon and ligament injuries occur annually in America, accounting for 30% of all musculoskeletal consultations, placing a heavy burden on the health care system.^[^ ^2^, ^3^ ^]^ However, it is hard to restore the structure and function of injured tendons to a native status because of low metabolism, poor blood vessel network, and hypocellularity.^[^ ^4^ ^]^ At the same time, activation and imbalance of the surrounding immune microenvironment form a vicious circle of enlarged inflammatory signals – aberrant repair of tissues, which further leads to continuous inflammatory infiltration and inadequate endogenous healing.^[^ ^5^ ^]^ Current clinical treatments, such as direct sutures, autologous transplantation, allogeneic transplantation, etc., have problems such as poor mechanical properties, immune rejection, donor site injury, and may not provide satisfactory long‐term clinical outcomes, such as retear, peritendinous adhesion and limited range of motion.^[^ ^6^, ^7^ ^]^

Tissue‐engineered scaffolds prepared by electrospinning techniques provide a good option for tendon repair.^[^ ^8^ ^]^ Preclinical studies demonstrated that scaffold with rational structure and material design could support tenocytes and regulate the microenvironment to promote tendon regeneration in a coordinated manner. To mimic the structure of the native tendon, various biophysical cues such as aligned arrangement,^[^ ^9^ ^]^ porous structure,^[^ ^10^ ^]^ groove design^[^ ^11^ ^]^ are provided to guide the interaction of cell‐biomaterial interfaces, such as promoting cell adhesion, proliferation, and tendon‐specific lineage differentiation.^[^ ^12^, ^13^ ^]^ In addition, the orientation structure is effective in regulating macrophage M2 polarization in vivo and reducing inflammatory activation after tendon injury.^[^ ^14^ ^]^ Adjustable degradation rates and excellent mechanical properties are also unavailable in extracellular matrix (ECM)‐derived scaffolds, providing stable mechanical support for critical early‐stage tissue repair.^[^ ^8^, ^15^, ^16^ ^]^

As the core of energy metabolism, mitochondria are involved in various biological processes.^[^ ^17^ ^]^ Rodeo et al. found that the mitochondria in degenerated tendons had a reduced ATP synthesis and expression of electron transport chain‐related genes (ATP5F1A, Fxn, oPA1, etc.). And the structural abnormalities of mitochondria were accompanied by a decrease in the biomechanical strength of tendons.^[^ ^18^ ^]^ Reactive oxygen species (ROS), as a byproduct of oxidative phosphorylation, is a key signaling molecule regulating the biological activity of tenocytes.^[^ ^19^ ^]^ Recent clinical studies reported that elevated levels of superoxide‐induced oxidative stress were associated with recurrent tears post‐arthroscopic rotator cuff repair.^[^ ^20^ ^]^ Meanwhile, excessive ROS also promotes macrophage M1 polarization in the early stage of tendon repair, expands the inflammatory response, and exacerbates the vicious cycle of imbalanced immune microenvironment‐abnormal tendon repair.^[^ ^21^, ^22^ ^]^ However, few empirical studies have explored the specific effects of oxidative stress‐mediated mitochondrial injury and immune microenvironment imbalances on tendon repair.

In recent years, inorganic nanomaterials with a variety of enzyme‐mimic activities, called nanozymes, have attracted significant attention in biomedicine. Compared with natural enzymes, nanozymes have the advantages of sustained activity, controllable release, multifunctional activity and low cost.^[^ ^23^ ^]^ They have been applied to various therapeutic fields such as nervous system diseases, diabetes, tumor and tissue repair.^[^ ^24^, ^25^, ^26^ ^]^ Cerium oxide nanoparticles (CeNPs), act as classical antioxidant nanozymes that exhibit superoxide dismutase (SOD) and catalase (CAT)‐mimic activities due to the easy switch of surface Ce (III)/Ce (IV) valency.^[^ ^23^, ^27^, ^28^ ^]^ In addition, nanoceria can scavenge hydroxyl radicals and nitric oxide radicals.^[^ ^29^, ^30^ ^]^ Based on satisfactory redox activity, nanoceria has shown broad application prospects in ROS scavenging‐related tissue repair, such as anti‐inflammation, antibacteria, angiogenesis, etc.^[^ ^31^, ^32^, ^33^, ^34^ ^]^ However, whether nanoceria could play a functional role in tendon repair remains unknown. Therefore, this study aimed to investigate whether nanoceria could maintain tenocytes' redox homeostasis and mitochondrial integrity and restore microenvironment balances under oxidative stress.

**Scheme 1:**
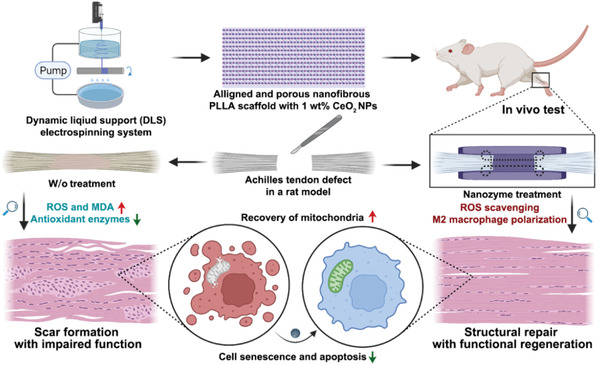
Schematic illustration of the therapeutic energy‐supporting enzyme‐mimic nanoscaffold for tendon regeneration. i) Anisotropic and porous PLLA nanofiber bundle scaffold with enhanced mechanical strength loaded with enzyme‐mimic cerium oxide nanoparticles (NBS@CeO) was prepared by dynamic liquid support (DLS) electrospinning system. ii) Persistent oxidative damage and mitochondrial dysfunction crippled the endogenous regenerative signaling and mediated the imbalanced immune microenvironment of injured tendon tissues and therefore led to scar formation with impaired function. iii) The implantation of the energy‐supporting nanozyme‐integrated scaffold (NBS@CeO) implemented ROS scavenging, recovered mitochondrial homeostasis and broke the vicious circle between repair signal abnormalities and immune signal imbalances. iv) The enhanced endogenous regenerative capacity and remodeling of the immune microenvironment promoted the regeneration of the ultrastructure of collagen fibers to recover mechanical properties and motor function.

In this work, we originally explored an energy‐supporting nanofiber scaffold based on a mitochondrial protection and immune remodeling strategy, named NBS@CeO, fabricated by dynamic liquid support (DLS) electrospinning system and functionalized with 1 wt% ceria nanozyme (**Scheme** **1**). The porous and anisotropic nanofiber bundle with enhanced mechanical properties could provide biophysical cues for tendon regeneration and successfully promote tendon repair. Meanwhile, nanoceria could effectively relieve oxidative stress, thus maintaining mitochondrial homeostasis and remodeling the immune microenvironment after tendon injury. Specifically, our in vitro and vivo experiments showed the tendon‐specific differentiation potential of TDSCs under oxidative stress was restored. Meanwhile, the pathological process of cell aging and apoptosis was simultaneously alleviated, which reflected the enlargement of endogenous regenerative signals. In the immune compartment, inflammatory signals were attenuated, manifesting as decreased levels of senescent‐related secretory phenotypic (SASP) and elevated levels of anti‐inflammatory cytokines. As a critical member in immunocytes, macrophages accelerated polarizing to pro‐resolving M2 phenotype and mediated a favorable platform for tissue repair. These results suggested that NBS@CeO could break the vicious circle between endogenous repair signal abnormalities and immune signal imbalances through mitochondrial homeostasis protection and immune microenvironment remodeling strategy. Furthermore, NBS@CeO successfully promoted the regeneration of the ultrastructure of collagen fibers to recover mechanical properties and motor function.

## 2 Results and Discussion

### 2.1 Evaluation of Oxidative Stress and Mitochondrial Destruction after Tendon Injury

**Figure 1:**
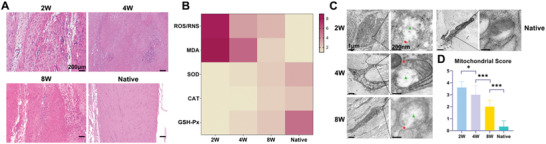
Persistent oxidative stress and mitochondrial structural damage in tendon tissue at various time points after injury. A) representative image of HE staining of native tendon and tendons at 2, 4, and 8 weeks postinjury (*n* = 3 per group). B) Heatmap of tendon oxidative stress (ROS, MDA) and antioxidant system (SOD, CAT, GSH‐Px) levels measured by ELISA postinjury (*n* = 3 per group). C,D) Representative images of mitochondrial structure and semiquantitative scoring of mitochondrial structure postinjury (*n* = 3 per group). Green triangle: mitochondrial swelling and vacuolization; Red star: mitochondrial membrane rupture. Data are presented as mean ± SD. *P < 0.05; ***P < 0.001.

Although the association of tendon injury with oxidative stress has been reported in clinical studies,^[^ ^20^ ^]^ in vivo evidence of continuous changes in oxidative damage and mitochondrial structural damage is still lacking. We established a rat model of Achilles tendon defect (ATD) and confirmed that structural tendon damage remained at least 8 weeks postoperatively (**Figure** **1**A). Total ROS/RNS and malondialdehyde (MDA) could reflect the severity of oxidative damage in injured tendons. SOD, CAT and glutathione peroxidase (GSH‐Px) consist of the main enzymes of antioxidant systems. Therefore, the levels of the above indicators were measured by ELISA and shown with a heatmap to evaluate redox homeostasis of injured tendons (Figure 1B). At 2 weeks postoperatively, the ROS/RNS and MDA levels were 8.31 and 8.93 times that of native tendons, respectively (*P* < 0.001). At 4 and 8 weeks postoperatively, the level of ROS/RNS and MDA, though reduced, were still significantly higher than the native control (*P* < 0.05). Meanwhile, natural antioxidant enzymes, mainly present in the mitochondria, suffered significant damage to their bioactivity. The average activity ratio of SOD, CAT, GSH‐Px to native tendons was 1:3.0, 1:2.2, and 1:5.2, respectively, at 2 weeks postoperatively (*P* < 0.001). At the 8 weeks postoperatively, the activity of these enzymes still did not return to native levels (*P* < 0.05). We then observed the mitochondrial ultrastructure with transmission electron microscopy (TEM) and found the morphology of mitochondria of injured tendons were swollen, and compartmentalized, even without a complete outer membrane (Figure 1C). Semi‐quantitative analysis of mitochondria was performed to evaluate the severity of structural damage (mitochondrial score: 2w, 3.6; 4w, 3.0; 8w, 2.0, respectively, *P* < 0.05). The above results showed continuous oxidative stress and mitochondrial damage during the repair process after tendon injury. Zhang et al. reported that the reduction of SOD activity and alterations in morphology and number of mitochondria also existed in a supraspinatus tendinopathy model.^[^ ^18^ ^]^ And the number of mitochondria and cristae gradually recovered after removing the injury. However, whether relieving oxidative stress is an effective mechanism for promoting tendon healing needs to explore further. Inspired by the fact that imbalance of the redox system and notable disruption of the mitochondrial structure after tendon injury, we developed a biomimetic fibrous scaffold with ROS scavenging capacity to explore the role of oxidative stress resistance and mitochondrial protection strategy in tendon healing.

### 2.2 Preparation and Characterization of NBS@CeO

**Figure 2:**
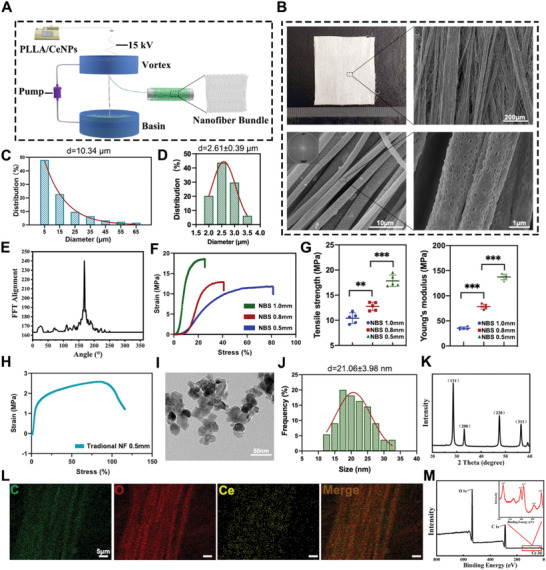
Fabrication and characterization of anisotropic and porous PLLA nanofiber bundle scaffold with enhanced mechanical strength. A) Schematic illustration of the fabrication of PLLA nanofiber bundle scaffold loaded with 1 wt% cerium oxide nanoparticles (CeNPs). B) Gross and SEM images of nanofiber bundle scaffold (NBS). C,D) Frequency distribution of the pore size and nanofiber diameter. E) FFT analysis of NBS. F,G) Strain–stress curve, tensile strength and Young's modulus of NBS fabricated with different thicknesses (*n* = 5 per group). H) Strain–stress curve of aligned nanofiber scaffold (NF) fabricated by traditional electrospinning technique. I–K) TEM, XRD, and the size distribution analysis of CeNPs. L,M) EDS and XPS analysis of NBS loaded with cerium oxide nanoparticles (NBS@CeO). Data are presented as mean ± SD. *P < 0.05; **P < 0.01; ***P < 0.001.

We successfully prepared a nanofiber bundle scaffold (NBS) with orientation arrangement and porous properties by dynamic liquid support (DLS) electrospinning system (**Figure** **2**A,B). The average fiber pore size of the scaffold was 10.34 µm and the porous fibers had an average diameter of 2.61 µm (Figure 2C,D). The loose and porous properties ensured a free exchange of nutrients between the microenvironment and tendon tissues. Figure 2B showed the fibers were aligned along the longitudinal direction of the scaffold. Meanwhile, FFT analysis showed that the fibers had a highly anisotropic structure (Figure 2E). The parallel fibers could guide the orderly arrangement of tenocytes and promote tendon‐lineage differentiation and deposition of ECMs.^[^ ^35^ ^]^ Recent studies reported that oriented fiber arrangement was advantageous in inhibiting macrophage M1 polarization and reducing tissue inflammation compared with disordered fibers.^[^ ^14^, ^35^ ^]^ All scaffolds emerged as typical stress‐strain curves for mechanical properties, as observed in Figure 2F,H. At the thickness of the scaffold was 0.5 mm, the breaking stress of the NBS group prepared by the DLS system (11.80 MPa) was significantly higher than that of the nanofiber scaffold (NF) group prepared by traditional electrospinning technique (2.58 MPa). Then, we made three different thicknesses of NBS and compared their mechanical properties (Figure 2F,G). The results demonstrated that NBS with a thickness of 1 mm had relatively superior tensile strength (17.84 MPa) and Young's modulus (137.41 MPa), providing more stable mechanical support for early‐stage tendon repair. Therefore, the NBS with a thickness of 1 mm was selected for following experiments.

**Figure 3:**
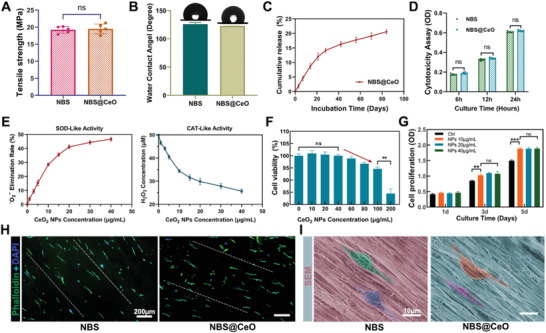
Characterization of physical, biochemical properties, and biocompatibility of nanofiber bundle scaffold loaded with cerium oxide nanoparticles. A,B) Tensile strength and water contact angle of NBS and NBS@CeO with the thickness of 1.0 mm (*n* = 5 per group). C) Release kinetics of NBS@CeO after incubation in PBS at 37 °C (*n* = 3 per group). D) Cytotoxicity assay of tendon‐derived stem cells (TDSCs) seeded on NBS and NBS@CeO after incubating for 6, 12, and 24 h (*n* = 5 per group). E) Enzyme‐mimic activity of gradient concentrations of CeNPs (*n* = 3 per group). F) Cell viability analysis of TDSCs under gradient concentrations of CeNPs tested by CCK‐8 after incubating for 24 h (*n* = 3 per group). G) Cell proliferation analysis of TDSCs under different concentrations of CeNPs tested by CCK‐8 after incubating for 1, 3, and 5 days (*n* = 3 per group). H,I) Immunofluorescence staining and SEM images of TDSCs on the surface of NBS and NBS@CeO after incubating for 24 h. Data are presented as mean ± SD. **P < 0.01; ***P < 0.001; ns, no statistical significance.

Cerium oxide nanoparticles (CeNPs, diameter ≈20 nm), were indicated by the TEM image and X‐ray diffraction (XRD) analysis (Figure 2I–K). As shown in Figure 2I, CeNPs were nearly spherical with an average diameter of 21.06 ± 3.98 nm (Figure 2J). XRD analysis (Figure 2K) demonstrated that CeNPs possessed a typical fluorite cubic structure. The X‐ray photoelectron spectroscopy (XPS) analysis (Figure S1, Supporting Information) indicated the coexistence of Ce^3+^ and Ce^4+^ on the surface of CeNPs at a ratio of 22.88:77.12, providing the structural basis for the recyclable ROS scavenging properties by the self‐redox reactions. Meanwhile, electron spinning resonance (ESR) spectroscopy was performed to further clarify the ROS scavenging performance of CeNPs. The hydroxyl radicals (·OH) and superoxide radicals (·O_2_^–^) generated by the Fenton reaction were detected by using 5,5′‐dimethylpyrroline‐1‐oxide (DMPO) as the spin trap agent. As shown in Figure S2A,C (Supporting Information), the appearance of the typical peaks in the ESR spectrum confirmed the presence of DMPO‐·OH and DMPO‐·O_2_^–^ adducts. Interestingly, a sharp decrease of relative signal intensity (Figure S2B,D, Supporting Information) was observed after adding CeNPs, indicating a drastic reduction in the amount of DMPO adducts and confirming the well‐performed ROS scavenging effect of CeNPs. Next, 1 wt% CeNPs were integrated to functionalize the scaffold for the following experiments. The energy dispersive spectroscopy (EDS) and XPS verified that the CeNPs were successfully integrated into the scaffold and dispersed in the direction of the fibers evenly (Figure 2L,M). There were no significant differences in the mechanical strength and water contact angle of NBS after the integration of CeNPs (**Figure** **3**A,B). Subsequently, we measured the release profile of CeNPs with inductively coupled plasma mass spectrometry (ICP‐MS), and the results showed that CeNPs were stably integrated into NBS with a release percentage of 20.6% after 84 days of incubation at 37 °C (Figure 3C). And there was no significant difference in cytotoxicity to TDSCs seeded on NBS and NBS@CeO (Figure 3D). The enzyme‐mimic activity of CeNPs, mainly SOD and CAT, was measured (Figure 3E). The results showed that CeNPs could catalyze superoxide anions and hydrogen peroxide in a concentration‐dependent manner, demonstrating their high ROS scavenging capability. Cell viability assay showed that CeNPs were not significantly cytotoxic when the concentration was lower than 40 µg mL^−1^. However, when the concentration reached 60 µg mL^−1^ or higher, the cell activity gradually decreased, showing the gradual increase of CeNPs in cytotoxicity (Figure 3F). Next, we found that lower concentrations of CeNPs had a promoting effect on cell proliferation by using CCK‐8 assay (Figure 3G). Finally, we explored the arrangement and morphology of TDSCs seeded on NBS and NBS@CeO (Figure 3H,I). The results showed that the TDSCs had a normal cell morphology on the scaffold and were arranged in parallel along with the orientation of the fibers, demonstrating the excellent biocompatibility of scaffolds and the biological role of their topology in guiding cell growth. Overall, a well‐designed CeNPs‐integrated NBS platform was successfully achieved. It is expected to provide adequate physical and biochemical cues for mitochondrial homeostasis maintenance and microenvironmental homeostasis restoration in tendon repair.

### 2.3 CeNPs Reduced Phenotypic Suppression on TDSCs Under Oxidative Stress in Vitro

**Figure 4:**
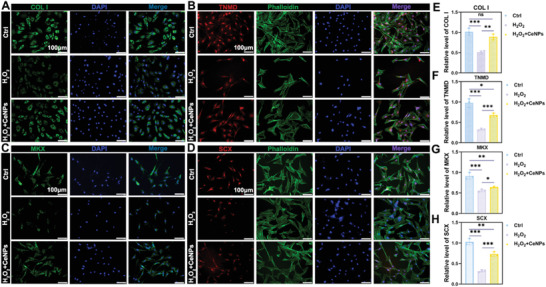
CeNPs alleviated the inhibition of tendon‐specific marker expression in TDSCs under oxidative stress in vitro. A–D) Immunofluorescence staining images of tendon markers (COL I, TNMD, MKX, SCX) of TDSCs. The nuclei were stained with DAPI and cytoskeletons were stained with phalloidin (*n* = 3 per group). E–H) Semiquantitative analysis of the relative fluorescent intensity of (A)–(D). Data are presented as mean ± SD. *P < 0.05; **P < 0.01; ***P < 0.001; ns, no statistical significance.

Based on previous studies, hydrogen peroxide (H_2_O_2_) could simulate an oxidative stress environment, suppress the proliferation and tenogenic differentiation of TDSCs.^[^ ^36^, ^37^ ^]^ Here, we used CeNPs with ROS scavenging capabilities to explore whether they could reduce the inhibitory effects of H_2_O_2_ on the tenogenic differentiation potential of TDSCs. The fresh medium culture alone served as the control group (Ctrl group). Type I collagen (COL I) is the main component of the extracellular matrix synthesized by TDSCs in normal tendons. Tenomodulin (TNMD) is a tendon‐specific marker, which could support the self‐renewal of TDSCs and suppress their senescence.^[^ ^38^ ^]^ Besides, Scleraxis (SCX) and Mohawk (MKX) are tenogenic transcription factors, which are essential for tendon‐lineage differentiation.^[^ ^39^ ^]^ Cellular immunofluorescence showed the supplement of 40 µg mL^−1^ CeNPs could alleviate the inhibition effect of COL I, TNMD, SCX, and MKX in TDSCs (COL I: *P* < 0.01; TNMD: *P* < 0.001; MKX: *P* < 0.05; SCX: *P* < 0.001) (**Figure** **4**A–D), compared with the H_2_O_2_ group, which may help improve endogenous tendon repair. Furthermore, we verified the expression of tendon‐specific markers at the transcriptional level. The qRT‐PCR results revealed significant differences among the three groups (Figure S3, Supporting Information). The relative RNA expression level of Col1a1 in the H_2_O_2_ + CeNPs group improved from 0.53 in the H_2_O_2_ group to 0.72 (*P* < 0.05), and the expression level of Tnmd increased from 0.43 in the H_2_O_2_ group to 0.70 (*P* < 0.05). Moreover, the expression level of two tenogenic transcription factors (Mkx and Scx) were also restored (*P* < 0.05). These results suggested that CeNPs could relieve the suppression of tenogenic phenotype of TDSCs under an oxidative stress environment, which may contribute to improving endogenous tendon healing.

### 2.4 CeNPs Reduced ROS Level, Maintained ΔΨm and ATP Synthesis of TDSCs in Vitro

**Figure 5:**
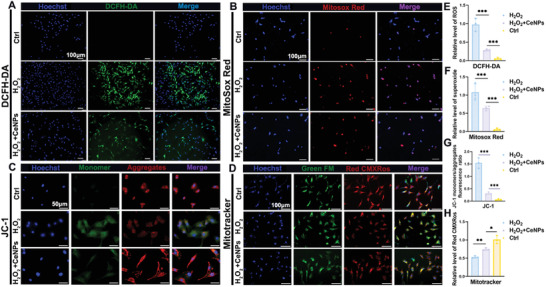
CeNPs alleviated H_2_O_2_‐induced oxidative stress and restored mitochondrial membrane potential of TDSCs in vitro. A,B) Immunofluorescence staining images of intracellular ROS and superoxide detected by DCFH‐DA probe and Mitosox Red indicator, respectively. C,D) Immunofluorescence staining images of mitochondrial membrane potential (ΔΨm) detected by JC‐1 probe and Mitotracker indicator, respectively. The nuclei were stained with Hoechst. E–H) Semiquantitative analysis of the relative fluorescent intensity of (A)–(D) (*n* = 3 per group). Data are presented as mean ± SD. *P < 0.05; **P < 0.01; ***P < 0.001.

**Figure 6:**
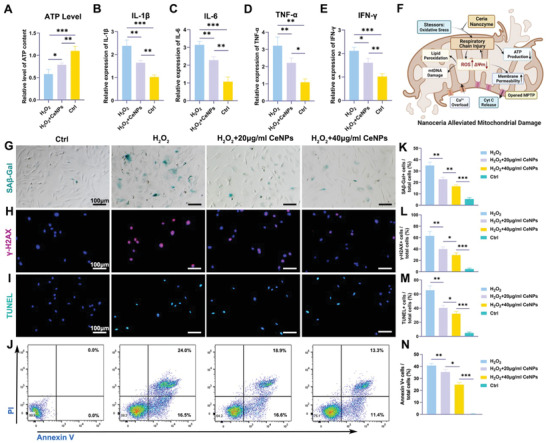
CeNPs maintained ATP levels, inhibited expression of inflammation‐related cytokines and reduced levels of cellular senescence and apoptosis of TDSCs in vitro. A) Estimation of cellular ATP level after 4 h of H_2_O_2_‐induced oxidative stress in the presence or absence of CeNPs using a bioluminescence detection kit (*n* = 3 per group). B–E) The proinflammatory cytokines (IL‐1𝛼, IL‐6, TNF‐𝛼, and IFN‐𝛾) gene expression of TDSCs measured by qRT‐PCR (*n* = 3 per group). F) Schematic representation of the effect of CeNPs on mitochondrial damage. G,H) SA*β*‐gal staining and immunofluorescence staining of DNA injury‐related protein *γ*‐H2AX of CeNPs‐treated TDSCs suffering from H_2_O_2_ stimulation. The blue cells are senescent. Scale Bar = 100 µm. I,J) Immunofluorescence staining of TUNEL and Annexin/PI‐labeled flow cytometry analysis of apoptotic TDSCs under oxidative stress environment. K–N) Semiquantitative analysis of the percentage of positive cells of (G)–(J) (*n* = 3 per group). Data are presented as mean ± SD. *P < 0.05; **P < 0.01; ***P < 0.001.

The total ROS and superoxide levels were detected by DCFH‐DA and Mitosox Red reagents, respectively. The relative DCFH‐DA fluorescence intensity in the H_2_O_2_+CeNPs group was significantly lower than in the H_2_O_2_ group (0.98 vs 0.28, *P* < 0.001) (**Figure** **5**A,E), reflecting the ROS level in TDSCs was reduced. Moreover, the level of superoxide was also significantly reduced (1.08 vs 0.64, *P* < 0.001) (Figure 5B,F). The massive accumulation of ROS, including superoxide will alter the membrane potential (ΔΨm) of the mitochondria, which in turn will lead to the energy crisis of the TDSCs, and even mediate the aging and apoptosis.^[^ ^40^, ^41^ ^]^ Therefore, JC‐1 probe was utilized to measure the ΔΨm. The immunofluorescence results showed notable JC‐1 monomers in the H_2_O_2_ group, indicating a significant decrease in ΔΨm. Interestingly, the co‐processing of TDSCs with CeNPs effectively stabilized the ΔΨm of mitochondria (Figure 5C,G). In addition, we used Mitrorakcer Red CMXRos, a ΔΨm indicator, to verify changes in membrane potential, and the results were consistent with the JC‐1 probe (Figure 5D,H). The stable production of ATP via F1‐F0 synthase is dependent on the maintenance of appropriate ΔΨm (–135 to –140 mV).^[^ ^42^ ^]^ We then evaluated the effect of ceria nanozyme on ATP levels with a luciferase detection kit. It was found that the ceria nanozyme successfully restored ATP production to some extent (**Figure** **6**A, H_2_O_2_+CeNPs vs H_2_O_2_, 0.78 vs 0.59, *P* < 0.05).

### 2.5 CeNPs Alleviated Inflammation Damage, Senescence, and Apoptotic Stress

The transition from limited ATP synthesis to ROS production leads to oxidative damage to mitochondria and other organelles, which could convert TDSCs to a pro‐inflammatory state, thereby initiating the senescence program.^[^ ^43^, ^44^ ^]^ Moreover, severe oxidative stress could lead to apoptosis of tenocytes, which is mediated by mitochondrial release of cytochrome C and triggering caspase‐3 activation.^[^ ^45^ ^]^ As the main pro‐inflammatory cytokines in SASP, the expression of IL‐1*β*, IL‐6, TNF‐*α*, and IFN‐*γ* was down‐regulated in H_2_O_2_ + CeNPs group, demonstrating a remission of the inflammatory state of the TDSCs (Figure 6B–E). Remarkably, senescence‐associated beta‐galactosidase (SA*β*‐gal) staining revealed that CeNPs inhibited the senescence of TDSCs (Figure 6G,K). Under safeguarding interference by CeNPs, DNA damage associated *γ*‐H2AX was also suppressed (Figure 6H,L). TUNEL staining and Annexin V/PI‐labeled flow cytometry analyzed the apoptotic TDSCs. The rate of TUNEL positive cells remarkably decreased (Figure 6I,M). And the Annexin V positive TDSCs also reduced to 24.7% in H_2_O_2_ + 40 µg mL^−1^ CeNPs group from 41.0% in H_2_O_2_ group (Figure 6J,N).

Overall, our in vitro experiments primarily demonstrated that CeNPs could effectively perform ROS clearance capabilities, and assist TDSCs in stabilizing ΔΨm and ATP production of mitochondria. Meanwhile, the tenogenic potential of TDSCs under oxidative stress was rescued, and the pathological process of inflammation, senescence and apoptosis of TDSCs was also alleviated. These cytological behaviors of TDSCs reflected that CeNPs possessed the potential to retrieve the reduction in endogenous repair signals under oxidative impairment. Given the encouraging protective properties of CeNPs, we further explored its effect in vivo after integration into nanofiber scaffolds.

### 2.6 NBS@CeO Reduced Oxidative Damage and Restored Mitochondrial Ultrastructure of Tendon in a Rat Model of ATD

**Figure 7:**
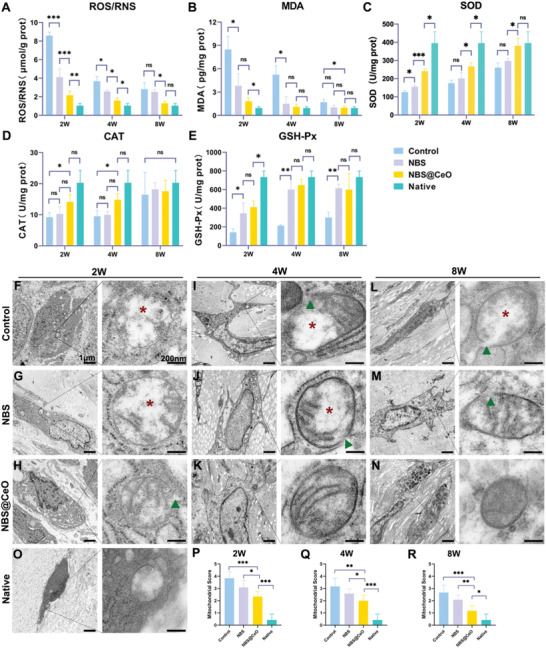
NBS@CeO alleviated the level of oxidative damage, restored antioxidant enzyme activity and mitochondrial ultrastructure at various time post tendon injury. A,B) The content of ROS/RNS and MDA of injured tendons measured by ELISA at 2 weeks, 4 weeks, and 8 weeks postinjury (*n* = 3 per group). C–E) Antioxidant enzyme system (SOD, CAT, GSH‐Px) of injured tendons measured by ELISA at 2 weeks, 4 weeks, and 8 weeks postinjury (*n* = 3 per group). F–H) Representative TEM images of mitochondrial ultrastructure of tendons at 2 weeks postinjury from control, NBS, and NBS@CeO group, respectively. I‐K Representative TEM images of mitochondrial ultrastructure of tendons at 4 weeks postinjury. L–N) Representative TEM images of mitochondrial ultrastructure of tendons at 8 weeks postinjury. O) Representative TEM images of mitochondrial ultrastructure from native tendon. P–R) Semiquantitative scoring of mitochondrial structure of 2, 4, and 8 weeks postinjury (*n* = 3 per group). Red star: mitochondrial swelling and vacuolization; Green triangle: mitochondrial membrane rupture. Data are presented as mean ± SD. *P < 0.05; **P < 0.01; ***P < 0.001; ns, no statistical significance.

**Figure 8:**
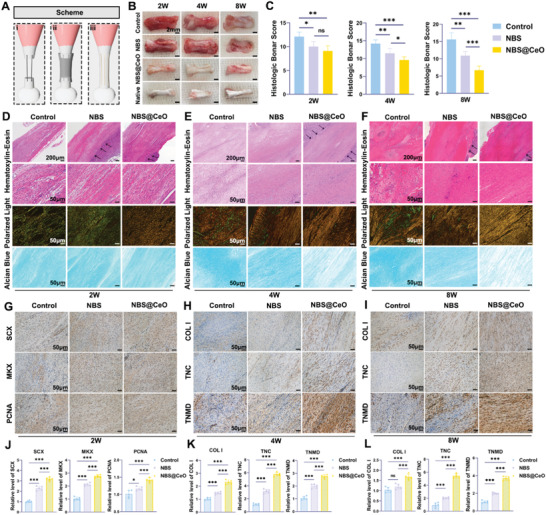
NBS@CeO promoted structural repair of injured tendons at macroscopic and histological levels at various time points postinjury. A) Schematic diagram of surgical procedures of the tendon defect rat model. B) Gross view of injured tendons from control, NBS, and NBS@CeO groups and native tendon at various time points postinjury. C) Semiquantitative Bonar scores for histological analysis of different groups at various time points postinjury (*n* = 5 per group). D–F) Representative images of H&E staining, polarized light microscopy (stained with Sirius Red), and Alcian blue staining of tendons from different groups at 2, 4, and 8 weeks postinjury. Black arrow: interfaces between the material and injured tendon. G–I) Representative images of immunohistochemical staining of tendon markers in early (SCX, MKX, PCNA) and mid‐advanced (COL I, TNMD, TNC) stages of repair in different groups at various time points after injury. J–L) Semiquantitative analysis of expression level of tendon marker of (F)–(H), respectively (*n* = 5 per group). Data are presented as mean ± SD. *P < 0.05; **P < 0.01; ***P < 0.001; ns, no statistical significance.

To verify the in vivo effect of NBS@CeO on boosting regenerative tendon healing, we established an Achilles tendon defect rat model, according to previously reported (Figure 8A).^[^ ^46^ ^]^ We first clarified the role of NBS@CeO in combating oxidative stress and protecting mitochondria. At different stages of tendon healing, the level of ROS/RNS, despite a continuous decline, dropped from 8.49 µmol g^−1^ at 2 weeks postoperatively to 2.50 µmol g^−1^ at 8 weeks postoperatively, still above normal (1.06 µmol g^−1^). Compared with the control group and the NBS group, the oxidative damage was continuously improved in the NBS@CeO group, and the difference was most evident at 2 weeks after surgery (*P* < 0.001) (**Figure** **7**A). MDA, a lipid peroxidation damage marker in mitochondria, also improved significantly in the NBS@CeO group (Figure 7B). The antioxidant enzyme is the main defensive line that scavenges ROS, protects mitochondrial contents and stabilizes the mitochondria membrane.^[^ ^47^ ^]^ SOD, forms the first line of defense against oxidative stress. Its activity in the control group was only 126.14 U mg^−1^ at 2 weeks postoperatively, and remained significantly lower than normal (395.18 U mg^−1^) until 8 weeks postoperatively (261.18 U mg^−1^). Interestingly, we found a significant recovery of SOD activity in the NBS@CeO group at 2 weeks postoperatively (240.89 U mg^−1^) and there was no significant difference from normal levels 8 weeks postoperatively (380.34 U mg^−1^) (Figure 7C). Inhibition of CAT mainly occurred at 2 and 4 weeks postoperatively (*P* < 0.05), and there was no significant difference between the control group activity and normal levels at 8 weeks postoperatively (Figure 7D). GSH‐Px, as the primary peroxidase, was continuously suppressed postinjury (2w, 141.60 U mg^−1^; 4w, 213.14 U mg^−1^; 8w, 299.59 U mg^−1^), while the NBS@CeO group improved its activity significantly (Figure 7E). Notably, the NBS group also alleviated oxidative injury and improved antioxidant enzyme activity postoperatively, which may be related to the fact that scaffolds can support tenocyte growth and differentiation in early‐stage of tendon repair, thereby enhancing regeneration signals and attenuating inflammation signal amplification, which broke the vicious cycle of tissue damage.

Ultrastructural changes in mitochondria closely reflected functional changes, such as mitochondrial swelling is one of the most important indicators of the opening of mitochondrial permeability transition pore.^[^ ^48^ ^]^ The control group showed that mitochondrial outer membrane rupture even lasted up to 8 weeks postoperatively, which led to the release of cytochrome C and triggered apoptosis or necrosis.^[^ ^49^ ^]^ Therefore, mitochondrial morphology assessment helps to assess the injury status of tendons and the protective effect of NBS@CeO. A semi‐quantitative grading system was used to examine the mitochondrial morphology. The mitochondrial score of NBS@CeO group (2.33 ± 0.41) was lower than control (3.83 ± 0.52) and NBS group (3.08 ± 0.58) at 2 weeks postoperatively (*P* < 0.05) (Figure 7F,G,H,P). At 4 weeks and 8 weeks postoperatively, the structure of part of mitochondria in the NBS@CeO group returned to complete without swelling or membrane distortion, exhibiting the lowest average score among the three groups (*P* < 0.05) (Figure 7I–N). Overall, our NBS@CeO reduced oxidative damage, improved antioxidant enzyme activities, and promoted mitochondrial structure recovery at 2 weeks, 4 weeks, and 8 weeks after tendon injury.

### 2.7 NBS@CeO Promoted Tendon Repair at Histological and Ultrastructural Levels in Vivo

At macroscopic level, tendons in the control group were forming granulation tissue in the early stages of repair (2 weeks and 4 weeks postoperatively), with a robust inflammatory response (**Figure** **8**B). In contrast, the NBS@CeO group had significantly lighter edema. At 8 weeks postoperatively, the control group were repaired with coarse and tough scar tissue, while the NBS@CeO group exhibited denser appearance close to the native tendon. At histological level, the semi‐quantitative revisited Bonar histological grading system was used to assess the tendon healing.^[^ ^50^, ^51^ ^]^ Hematoxylin and eosin staining (H&E staining) was used to assess cell morphology, cellularity, angiogenesis, and collagen arrangement. In addition, ectopic ossification and adipocyte infiltration were also assessed by H&E staining. Images from polarized light microscopy could assist in the assessment of collagen fiber density and arrangement. Alcian blue staining could reflect the secretion of ground substance and indicate fibrocartilaginous tissue. The results indicated that the collagen fibers in the control group were most loose and disorganized at 2 weeks postoperatively, with persistent inflammatory cell infiltration and neovascularization (Bonar score = 12.1) (Figure 8D). At 8 weeks postoperatively, a part of tenocytes showed chondrocyte‐like changes, and the strongly positive of Alcian blue staining also suggested the formation of fibrocartilaginous tissue (Bonar score = 15.6) (Figure 8F). It is worth noting that the tendons of NBS and NBS@CeO groups had a significantly more oriented arrangement and higher density at 2 weeks after surgery. As the repair progressed, the inflammation in NBS@CeO group gradually decreased, the density of collagen fiber increased significantly, and there was no ossified tissue or adipocyte infiltration, suggesting excellent histological repair (Bonar score = 6.7). Immunohistochemical results suggested that tendon‐specific transcription factors SCX and MKX are significantly upregulated in NBS@CeO in the early stage of tendon repair, showing an enhancement in initiation of tenogenesis (Figure 8G,J).^[^ ^52^ ^]^ Proliferating cell nuclear antigen (PCNA), as a marker of cell proliferation,^[^ ^53^ ^]^ was also upregulated in NBS@CeO group. In the middle and advanced stage of tendon repair, the delivery of ceria nanozyme yielded more mature tendon‐specific ECM, which was reflected by the significant upregulation of COL I, tenascin‐C (TNC) and TNMD, possibly due to the protective effect of nanozymes on endogenous tendon cells (Figure 8H,I,K,L). It was worth noting that NBS also promoted the expression of tendon‐specific markers postoperatively. These results illustrated the highly instructive biophysical cues of NBS could successfully guide tenogenic differentiation and thus promote endogenous tendon repair.^[^ ^12^ ^]^

**Figure 9:**
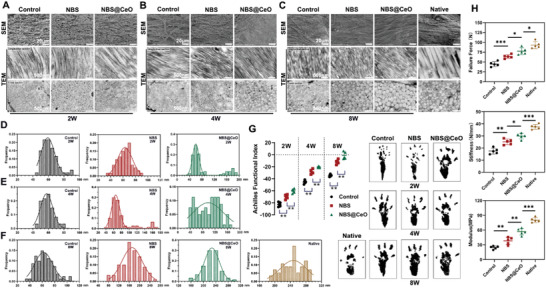
NBS@CeO improved ultrastructural morphology and promoted functional regeneration of injured tendons at various time points postinjury. A–C) Representative SEM and TEM images of injured tendons from different groups and native tendons at various time points postinjury. The longitudinal and transverse sections of the ultrastructure morphology of tendons were displayed under TEM observation (*n* = 3 per group). D–F) Representative analysis of the distribution of collagen fibril diameters (nm) of tendons from (A) to (C), respectively. G) Footprints and Achilles Functional Index (AFI) of rats from different groups at various time points postinjury (*n* = 4 per group). H) The biomechanical properties (failure force, stiffness, and modulus) of injured tendons were evaluated at 8 weeks after tendon injury (*n* = 5 per group). Data are presented as mean ± SD. *P < 0.05; **P < 0.01; ***P < 0.001.

At ultrastructural level, scanning electron microscopy (SEM) and TEM were utilized to evaluate the alignment, density, and diameters of collagen fibers, which were predictors of the mechanical properties of healing tendons. It was revealed that the collagen fibers of the control group were in loose and chaotic arrangements and had reduced diameter of 59.22 ± 8.60 nm (**Figure** **9**A,D) at 2 weeks postoperatively. Despite the increased density of collagen fibrils, the average diameter of the control group did not improve significantly and the microfibrils (<100 nm) were still dominant at 8 weeks postoperatively (62.33 ± 14.29 nm). And the diameter of NBS group was 171.10 ± 38.68 nm at 8 weeks postoperatively, indicating that NBS had a promoting effect on the conversion of type III collagen to type I collagen (Figure 9F). Meanwhile, we observed a gradual improvement in collagen diameter and density of NBS@CeO group. The regenerated tendons of NBS@CeO group had more orderly aligned and denser collagen fibers with a larger diameter of 226.40 ± 28.41 nm than the control and NBS groups at 8 weeks postoperatively. However, their collagen fibers were still smaller in diameter (247.70 ± 33.84 nm) than native tendons, which may reflect that their biomechanical properties still not recovered to the native level.

### 2.8 NBS@CeO Improved Mechanical Properties and Motor Function of Tendon in Vivo

In addition to evaluating tendon structure, the biomechanical properties are also crucial indicators for the recovery of healing tendons. We analyzed the biomechanical properties (failure force, stiffness, and modulus) of tendons at 8 weeks postoperatively based on force‐displacement curves using the Instron universal testing system. The failure force of Achilles tendons from control group, NBS group was 44.80 ± 6.30 N, 64.00 ± 4.74 N, respectively, which were significantly lower than that of NBS@CeO group (77.60 ± 8.71 N) and native tendons (93.00 ± 8.63 N) (Figure 9H). The stiffness of the NBS@CeO group (29.90 ± 2.31 N mm^−1^) was higher than that of the NBS group (25.14 ± 2.31 N mm^−1^) and control group (17.56 ± 2.45 N mm^−1^), but still lower when compared with native tendons (37.48 ± 2.01 N mm^−1^). The tensile modulus of the control group (24.52 ± 3.29 MPa)was significantly lower than the NBS group (38.08 ± 7.16 MPa) and NBS@CeO group (56.62 ± 6.80 MPa). Although the delivery of ceria nanozyme yielded the highest values among the three groups, it was still lower than the native tendons (80.20 ± 4.98 MPa, *P* < 0.05). At 2, 4, and 8 weeks postoperatively, the Achilles Functional Index (AFI) was recorded and analyzed in order to evaluate the functional performance of repaired tendon. According to Figure 9G, the hind paw prints of the repaired side in control group were considerably longer and narrower than the native side, and their AFI value (−82.67 ± 3.63) was most negative, which represented most serious hypomotility in all groups postoperatively. With the increased time of tendon healing, the control group achieved partial recovery at 4 weeks and 8 weeks postoperatively. Notably, the NBS@CeO group showed distinctly higher AFI value than NBS and control group postoperatively. At 4 weeks postoperatively, the AFI value of NBS@CeO group recovered to −20.51 ± 1.18, which was remarkably higher than that of 2 weeks postoperatively (−60.17± 3.74). And at 8 weeks postoperatively, the AFI value of NBS@CeO group approached the native level (−1.38 ± 6.00), indicating satisfactory repair performance.

### 2.9 NBS@CeO Alleviated Senescence and Apoptotic stress, Promoted M2 Polarization of Macrophages and Reduced inflammation damage in Vivo

**Figure 10:**
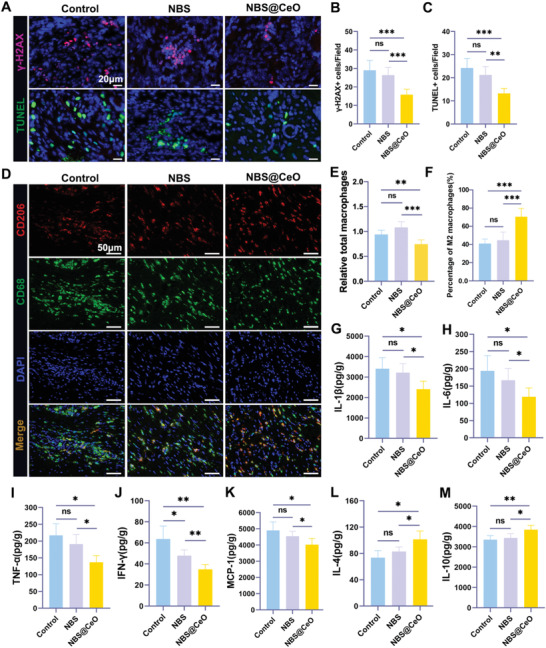
NBS@CeO alleviated senescence and apoptotic stress, promoted M2 phenotype polarization of macrophages and reduced inflammation damage. A) Representative immunofluorescence staining of DNA injury‐related protein *γ*‐H2AX and TUNEL of injured tendons from different groups at 2 weeks postinjury. B,C) Semiquantitative analysis of the number of *γ*‐H2AX positive and TUNEL positive cells in each field of view (*n* = 5 per group). D) Representative immunofluorescent images of total and M2 polarization macrophages of injured tendons from different groups at 2 weeks postinjury stained by CD68 and CD206, respectively. E,F) Semiquantitative analysis of the number of CD68 positive macrophages and the ratio of CD206/CD68 positive cells in each field of view (*n* = 5 per group). G–M) SASP‐related cytokine expression was detected by ELISA, including IL‐1*β* G), IL‐6 H), TNF‐*α* I), IFN‐*γ* J), MCP‐1 K), IL‐4 L), IL‐10 M) (*n* = 5 per group). Data are presented as mean ± SD. *P < 0.05; **P < 0.01; ***P < 0.001; ns, no statistical significance.

After confirming that NBS@CeO could promote tendon structural and functional regeneration through mitochondrial protection, we further explored the senescence and apoptosis stress and state of immune microenvironment in healing tendons. Pathologically, oxidative stress mediates inflammatory activation, including activation of M1 macrophages and unrestricted secretion of pro‐inflammatory factors.^[^ ^43^, ^54^ ^]^ Meanwhile, M1 macrophage polarization and persistent inflammation initiate senescence programs.^[^ ^44^, ^55^ ^]^ And SASPs, including IL‐1*α*, IL‐6, and IFN‐*γ* could induce M1 polarization of macrophages, thereby enhancing the early inflammatory response.^[^ ^56^ ^]^ Taken together, these processes constitute a vicious cycle between senescence and inflammation, impairing tendon healing. Based on our results, it was found that the NBS@CeO could alleviate tissue inflammation in the early stage of tendon healing (at 2 weeks postoperatively), which was reflected by the decrease of *γ*‐H2AX positive cells (**Figure** **10**A,C), CD68 positive cells (Figure 10D) and SASP secretion levels (Figure 10G–J). These results illustrated that ceria nanozyme could effectively break the vicious cycle of inflammation and cell senescence. Moreover, oxidative stress‐mediated cell apoptosis was also significantly relieved, which was consistent with our results of in vitro studies (Figure 10A,D). It was worth noting that M2 phenotype macrophages dominated in NBS@CeO group, which favored promoting inflammation regression, tenocyte proliferation, and ECM deposition.^[^ ^57^ ^]^ Snedeker et al. reported that different topological cues could drive different tendon immune responses in vivo. And scaffold with aligned fibers could significantly reduce NF‐*κ*B‐mediated “pro‐inflammatory activation” and facilitate macrophage M2 phenotype polarization.^[^ ^14^ ^]^ Similarly, our results showed that the proportion of M2 macrophages and the concentration of pro‐inflammatory cytokines were also improved in NBS group, although there were no significant differences. These results reflected the ability of NBS@CeO to enhance tendon regeneration through mitigating oxidative stress, regulating immune microenvironment and amplifying the signal of endogenous regeneration.

**Figure 11:**
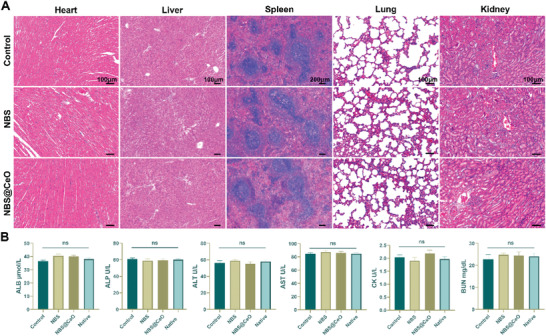
Verification of biological safety of NBS and NBS@CeO in vivo. A) Morphology of the major function organ (heart, liver, spleen, lung, and kidney) from control, NBS, and NBS@CeO groups at 8 weeks postoperatively (*n* = 3 per group). B) Blood biochemistry tests at 8 weeks postoperatively (*n* = 3 per group). ALB, albumin; ALP, alkaline phosphatase; ALT, alanine aminotransferase; AST, aspartate transaminase; CK, creatine kinase; BUN, blood urea nitrogen. Data are presented as mean ± SD. ns, no statistical significance.

Finally, we confirmed the satisfactory biocompatibility of our NBS and NBS@CeO scaffold in vivo. It is an everlasting matter of the biocompatibility of the nanomaterials. The reinforcing nanomaterial may release toxic compounds and accumulate in animal organs when the substrate is degraded. It is expected that supplemented nanomaterials could provide satisfactory biological properties without significant toxicity in vivo.^[^ ^58^ ^]^ Cerium oxide nanoparticles cater to these necessities with outstanding ROS scavenging properties and biocompatibility. The primary function organs, including the heart, liver, spleen, lung, and kidney, exhibited no structural abnormality at 8 weeks postimplantation of different scaffolds (**Figure** **11**A). Meanwhile, there were no significant differences in the main biochemical indicators of liver and kidney function (Figure 11B). Hence, our ceria nanozyme integrated scaffold could function as a bio‐friendly and superior bioactive nanomaterial for tendon regeneration.

## 3 Conclusions

Inspired by the fact that excessive oxidative stress impairs tendon repair, we explored an energy‐supporting enzyme‐mimic nanofiber scaffold‐based mitochondria protection and microenvironment remodeling strategy. Our ceria nanozyme integrated nanofiber bundle scaffold, named NBS@CeO, was anisotropic and porous with enhanced mechanical properties prepared by DLS electrospinning system. First, NBS@CeO could provide biophysical cues for tendon repair. Second, NBS@CeO relieved oxidative damage and maintained mitochondrial homeostasis. Furthermore, NBS@CeO mediated the rebalance of endogenous repair signaling and dysregulated immune microenvironment by alleviating senescence and apoptosis of TDSCs, downregulating the secretion of SASP, and inducing macrophage M2 polarization. Finally, the enhanced regenerative capacity and remodeling of the microenvironment empowered by NBS@CeO promoted the regeneration of the ultrastructure of collagen fibers to recover mechanical properties and motor function. Therefore, our NBS@CeO was observed to address the challenge of poor tendon healing from a novel pathophysiological perspective. Overall, this innovative strategy focuses on the biological role of ceria nanozyme and highlights the unexplored role of NBS@CeO in mitochondrial protection and immunomodulation in the field of tendon repair for the first time. It brings an in‐depth insight into biomimetic tendon scaffolds integrated with nanozymes, and provides a promising direction for exploring the translation to clinical applications.

## 4 Experimental Section

### Materials

Poly‐l‐lactic‐acid (PLLA) was purchased from Jinan Daigang Biomaterial with *M*_w_ = 21 KDa. Dimethyl Formamide (DMF) and dimethyl sulfoxide (DMSO) from Shanghai Chemical Reagents were used as the solvent for PLLA. Cerium oxide nanoparticles (CeNPs) were purchased from Rhawn Reagent (China).

### Fabrication of CeO_2_ Nanoparticle‐Loaded Nanofiber Bundle Scaffold

The nanofiber bundle scaffold (NBS) was fabricated using a dynamic liquid electrospinning machine as shown in Figure 2A. Polymer solution with a concentration of 12 wt% was prepared by dissolving PLLA into a mixture of DCM and DMF (2:1, v/v), followed by dispersing cerium oxide nanoparticles (CeNPs) with 1 wt% to acquire the electrospinning solution. Then, a 10 mL syringe with a blunt needle was loaded with the resultant solution and fed at a rate of 1 mL h^−1^. A 15 kV voltage was applied to a spinneret 15 cm away from the collecting target. Nanofiber bundles deposited in a water vortex were collected by spinning a drum with a speed of 100 rpm. Following the above electrospinning parameters, the collection device is turned into a solid plate to obtain the nanofiber scaffold (NF).

### Characterization of CeO_2_ Nanoparticle and CeNPs‐Loaded Nanofiber Bundle Scaffold

Freeze‐dried scaffold samples were sputter‐coated with gold, and their microstructure and nanofiber morphology were studied by SEM (TM‐3000, Hitachi, Japan). The diameter distribution of the fibers was measured by image analysis software (Image J). Then SEM images with the 2D Fast Fourier transform (FFT) function of Image J were analyzed to quantify fiber arrangement of the scaffold. The pore size distribution was measured by CFP‐1100‐AI Capillary Flow Porometer (PorousMaterials Inc, USA). The distribution and diameter of CeNPs were observed by transmission electron microscopy (TEM, JEM‐2100, Japan). Further, the arrangement of Ce element on the nanofiber bundles is analyzed by EDS (JSM‐7500F, China). The crystalline phase of CeNPs element and chemical structure of the scaffold was tested by XRD (Rigaku Corporation, Smartlab9kw, Japan) and XPS (Thermo Fisher, Escalab 250Xi, USA). The ESR (Bruker A300 spectrometer, USA) was conducted to examine the ROS scavenging properties of CeNPs. Briefly, the Fenton reaction (70 µL, 0.735 × 10^−3^ m FeSO_4_ and 25 µL, 0.315 × 10^−3^ m H_2_O_2_) was used to generate hydroxyl radicals (·OH) and superoxide radicals (·O_2_^–^), and 5,5′‐dimethylpyrroline‐1‐oxide (DMPO) was selected as the spin trap agent (5 µL, 98%) in the ESR spectrum to evaluate the capability of CeNPs in scavenging ROS. The change of relative peak intensity of ESR spectrum of the DMPO‐·OH and DMPO‐·O_2_^–^ adducts reflected the effect of CeNPs (100 µL, 1 × 10^−3^ m) on free radical elimination. Mechanical properties of the scaffold were obtained by a material testing machine (Wenzhou Darong Textile Instrument Co., Ltd., China). All samples with a dimension of 30 × 10 mm were stretched at a constant cross‐head speed of 100 mm min^−1^. Surface wettability of nanofiber bundle membranes was evaluated by measuring the water contact angle (OCA 15EC, DataPhysics Instrument, Germany). The release profile of CeNPs from NBS@CeO was measured by inductively coupled plasma mass spectrometry (ICP‐MS, Agilent 7500 series, USA) as previously reported.^[^ ^59^ ^]^ Briefly, after weighed accurately, the samples were immersed in 10 mL PBS (pH = 7.4), and incubated in a shaker incubator at 100 rpm at 37 °C. Fresh PBS was added at regular intervals to replace the solution removed for analysis. At 418 nm, the cerium emission line, the ceria concentration was determined, as well as the accumulated release percentage.

The enzyme‐mimic activity of CeNPs was measured as previously published.^[^ ^60^ ^]^ Briefly, the SOD‐mimic activity of CeNPs was examined by utilizing the total superoxide dismutase assay Kit (Biotech, China) to evaluate the ·O_2_^–^ scavenging activity. 20 µL of CeNPs dispersion at different concentrations (0, 1, 3, 5, 10, 15, 20, 30, 40 µg mL^−1^) was incubated with assay reagent into a 96‐well plate for 30 min. The absorbance at 560 nm was recorded using a Multiskan GO microplate reader (Thermo Fisher Scientific, USA), and calculated the inhibition rate following the manufacturer's instructions. Then the catalase‐mimic activity of CeNPs was measured with Catalase Assay Kit (Biotech, China). After drawing the H_2_O_2_ standard curve, a blank control and a sample solution were prepared, and were reacted with the assay agent at 25 °C. Then, a stop and color‐developing solution were added and incubated at 25 °C for 15 min. The absorbance at 520 nm was measured by a Multiskan GO microplate reader. Finally, the catalase‐mimicking activity of CeNPs was calculated following the manufacturer's procedures.

### Cell Culture, Toxicity and Proliferation Assay

Primary TDSCs were isolated from 6 to 8 weeks old rats following the established method.^[^ ^61^ ^]^ In brief, the harvested Achilles tendon was minced and digested in 3 mg mL^−1^ collagenase I (Servicebio, China) and 4 mg mL^−1^ dispase (Roche, USA) in a shake incubator at 37 °C for 2 h. After filtrating and centrifuging, the single‐cell suspensions were cultured in *α*‐MEM (Hyclone, USA) supplemented with 10% fetal bovine serum (Gibco, USA) and 100 U mL^−1^ penicillin/streptomycin (Servicebio, China). When the cell confluence arrived 80%, the cells were trypsinized, centrifuged, resuspended in complete fresh medium as passage 1, and incubated in a constant temperature incubator with 5% CO_2_ at 37 °C. The complete medium was refreshed every 2–3 days.

To examine the toxicity of NBS@CeO and NBS, the scaffolds were cut into small pieces that fit the size of six‐well culture plate. Next, sterilizing the samples by ultraviolet light and alcohol for 30 min. Cell counting kit‐8 (CCK‐8, Servicebio, China) was used for cytotoxicity assay. TDSCs viability was evaluated at 6, 12, and 24 h after being seeded on scaffolds. Then, the CCK‐8 working solution was added and reacted with TDSCs for 4 h. Using the Multiskan GO microplate reader, optical density (OD) value was measured at 450 nm wavelength. Similarly, the cytotoxicity of different concentrations of CeNPs (0, 5, 10, 20, 40, 60, 80, 100, 200 µg mL^−1^) was measured by CCK‐8 after incubating with CeNPs for 6 h. And cell proliferation assay was also tested by CCK‐8 under different concentrations of CeNPs (0, 10, 20, 40 µg mL^−1^). After culturing for 1, 3, and 5 days, the OD value was measured to evaluate the proliferation rate of TDSCs.

### Cell Alignment and Morphology on the CeNPs‐Loaded Nanofiber Bundle Scaffold

3 × 10^4^ cm^–2^ TDSCs were seeded on the NBS and NBS@CeO in a six‐well culture plate. After culturing for 3 days, TDSCs were fixed with 4% paraformaldehyde (PFA) and stained with 4,6‐diamidino‐2 phenylindole (DAPI, 1:500, Gibco, USA) and FITC‐Phalloidin (1:1000, Thermo Scientific, USA) for alignment assessment under DMi8 fluorescent microscopy (Leica, Germany). For morphology evaluation, TDSCs were fixed in 2.5% glutaraldehyde (Sigma Aldrich, USA) at 4 °C overnight, dehydrated by serial ethanol and sprayed with gold to enhance electroconductivity. Then the samples were observed and recorded under SEM (SU8010, Hitachi, Japan).

### Cell Immunofluorescence of Tendon‐specific Markers Under Oxidative Stress Model in Vitro

TDSCs were pretreated with 40 µg mL^−1^ CeNPs for 6 h. To mimic the oxidative stress microenvironment, TDSCs were exposed to 1 × 10^−4^ m H_2_O_2_ (Sigma‐Aldrich, USA) in serum‐free *α*‐MEM for 4 h. Then refreshed with *α*‐MEM complete medium and cultured for 3 days, TDSCs were washed with PBS twice and fixed with 4% PFA. After blocked by 1% BSA (Servicebio, China), samples were incubated in primary antibodies (Abcam, USA) were anti‐COL I (1:200), anti‐TNMD (1:200), anti‐MKX (1:200) anti‐SCX (1:200) overnight at 4 °C. Then the samples were washed with PBS, FITC‐conjugated and TRITC‐conjugated secondary antibodies (1:200, Abcam, USA) were added for reaction about 1 h at ambient temperature. Finally, the nuclei were stained with DAPI for 5 min. Each slide was photographed using DMi8 fluorescent microscopy.

### Detection of ROS Level, Mitochondria Membrane Potential, and ATP Production

ROS Assay Kit (Beyotime, China) and MitoSox Mitochondrial Superoxide Indicator (Thermo Scientific, USA) were used to detect ROS and superoxide of TDSCs according to instructions. And JC‐1 (Beyotime, China) and Mito‐Tracker Green/Red CMxRos (Yeasen, China) were used to detect the mitochondrial membrane potential of TDSCs. Briefly, after TDSCs were exposed to 100 × 10^−6^ m H_2_O_2_ in serum‐free *α*‐MEM for 4 h, the probe was mixed with serum‐free culture medium and diluted to work concentration. After incubating for 20 min, the Hoechst staining solution (1:100, Servicebio, China) was used to stain the nuclei. Then the cell plates were observed and the images were captured using DMi8 fluorescent microscopy. The cellular ATP levels were determined luminometrically using ATP assay reagent (Beyotime, China) according to previously mentioned protocol.^[^ ^40^ ^]^ Briefly, the TDSCs lysates were collected and used to determine ATP content. The resulting luminescence was measured by Varioskan LUX multimode plate reader (Thermo Scientific, USA). Finally, ATP concentrations were calculated according to a standard curve.

### Cell Senescence and Apoptosis

After exposed to 2 × 10^−4^ m H_2_O_2_ in serum‐free *α*‐MEM for 4 h, the senescence of TDSCs was investigated by SA*β*‐gal staining and *γ*‐H2AX immunofluorescence staining and following manufacturer instructions (Cell Signaling Technology, USA).^[^ ^62^ ^]^ TUNEL staining kit (Servicebio, China) was used to detect cell apoptosis according to instructions. Briefly, after the cells were fixed and permeabilized with Proteinase K, TUNEL reaction buffer was added for reaction for 60 min. Subsequently, the slides were observed and recorded using DMi8 fluorescent microscopy. The apoptotic status of cells was analyzed by flow cytometer and visualization of flow cytometry data was performed using FlowJo 10.9.1 software. Briefly, after coincubation with H_2_O_2_, TDSCs were washed with PBS gently, and then followed by incubation with Annexin V‐FITC and propidium iodide (PI) staining solution for 20 min away from light. Then the samples were analyzed by flow cytometer (CytoFLEX, USA) immediately, and data were analyzed by FlowJo software.

### Evaluation of Tendon‐Specific Markers and Proinflammatory Cytokines by qRT‐PCR

Total RNA from TDSCs was extracted using RNA Purification Kit (EZBiosience, USA) according to instructions. Briefly, the samples were lysed, homogenized, and centrifuged for RNA binding. After washed and centrifuged again, the purified RNA was eluted. Then the cDNA template was obtained by Reverse Transcription Mix II (EZBiosience, USA). After preparing the reaction system using the qPCR master mix (EZBiosience, USA), the amplification program was performed in the QuantStudio 6 and 7 Flex Real‐Time PCR Systems (Thermo Fisher Scientific, USA), and Ct values were obtained. Gapdh served as an internal reference. The primer sequences (forward 5′–3′) included Col1a1: GACGCATGGCCAAGAAGACAT, Tnmd: GACCTATGGCATGGAGCACAC, Mkx: TTACAAGCACCGTGACAACCC, Scx: CAACGTGCTACTGGTGGGTGA, IL‐1*β*: CACCTCTCAAGCAGAGCACAG, IL‐6: CGAAAGTCAACTCCATCTGCC, TNF*α*: TGCCTCAGCCTCTTCTCATTCCT, IFN‐*γ*: ATGAAATATACAAGTTATATG. Gapdh: CTGGAGAAACCTGCCAAGTATG.

### Animal Surgery and Therapy Setup

All the procedures regarding animal maintenance and experiments were approved by the Institutional Animal Care and Use Committee (IACUC) of the Shanghai Jiaotong University affiliated Shanghai Sixth People's Hospital (DWLL2022‐0004). Each SD rat weighing between 200 and 220 g was randomly assigned to one of three groups (*n*  =  30 per group). After anesthetized with 0.5% amobarbital sodium by intraperitoneal injection, the hind legs of rats were disinfected and shaved. A 5 × 1 mm^2^ rectangular full‐thickness defect was constructed to the left Achilles tendon. After operation, surgical limbs were immobilized for 1 week. Rats who underwent the surgical procedure were randomly divided into three groups: 1) control group: no treatment after above‐mentioned surgical procedures, 2) NBS group: implantation with NBS at the Achilles tendon defect site, and 3) NBS@CeO group: implantation with NBS@CeO at the Achilles tendon defect site. An Ethicon 6‐0 suture was used to attach the scaffold to the broken ends of the tendon defect. Upon sacrifice, the repaired tendons were harvested at 2, 4, and 8 weeks for macroscopy, histology, immunochemistry, immunofluorescence, SEM and TEM images, ELISA, biomechanics and gait analysis to assess the structural and functional recovery.

### Oxidative Stress Level, Antioxidative Capabilities, and Inflammatory‐Related Cytokines Quantified by ELISA

After obtaining the tissue lysates, the ROS/RNS level was measured using the OxiSelect ROS/RNS Assay Kit (Cell Biolabs, USA) following manufacturer's instructions. ELISA Kits also measured the MDA, SOD, CAT, GSH‐Px concentrations in accordance with standard instructions (Njjcbio, China). According to the manufacturer's manual, quantification of inflammatory‐related cytokines was performed using ELISA Kits (Invitrogen, USA).

### Macroscopic Evaluation of Repaired Tendons

At 2, 4, and 8 weeks postoperatively, the Achilles tendons were fully exposed and the full‐length tendon was harvested. Then, the tendons were placed on wet gauze and photographed by a camera (Nikon, Japan).

### Histological Staining and Biocompatibility Evaluation, Immunohistochemistry, and Immunofluorescence

The harvested samples were fixed immediately in 10% formalin for 24 h, dehydrated with gradient alcohols before embedded in paraffin. Then histological sections were prepared with a microtome with a thickness of 5 µm. A standard procedure for staining soft tissue with H&E, Alcian Blue, and Sirius Red was performed to examine the general histological structure of the tissues according to standard procedures. Major functioning organs including heart, liver, spleen, lung, and kidney were also observed by H&E staining to evaluate the biocompatibility of the scaffolds. Polarized light microscopy after Sirius Red staining was used to analyze collagen density and arrangement. Semiquantitative analysis of revisited Bonar score was conducted as published previously.^[^ ^50^ ^]^ The protein expression level in repaired tendons was analyzed by immunohistochemistry or immunofluorescence. Immunohistochemistry was performed according to the established standard procedures. In brief, the sections were deparaffinized, blocked and incubated with primary antibodies (Abcam, USA) including anti‐SCX, anti‐MKX, anti‐PCNA, anti‐COL I, anti‐TNC, and anti‐TNMD overnight at 4 °C, subsequently incubating with horseradish‐peroxidase‐conjugated secondary antibodies (Abcam, USA). For immunofluorescence, the sections were incubated with primary antibodies (Abcam, USA), including anti‐*γ*H2AX, anti‐CD68, and anti‐CD206, followed by incubating with FITC or TRITC‐conjugated secondary antibodies for 1 h in a room temperature. The TUNEL assay was performed according to previously described, aiming to evaluate the levels of cell apoptosis.^[^ ^63^ ^]^ Nuclei were stained with DAPI. The sections were observed using DMi8 fluorescent microscopy. For blood biochemistry tests, the blood from the caudal vein of rats was collected and used for assessing liver and kidney function at 8 weeks postoperatively. Briefly, the samples were centrifuged at 15 000 × *g* for 15 min, then the serum was carefully separated and placed in anticoagulant tubes for future tests on chemistry analyzer.

### Scanning Electron Microscopy and Transmission Electron Microscopy

For SEM, 1 × 1 mm excised samples were double‐fixed with 2.0% glutaraldehyde for 24 h and 2% osmium tetroxide for 2 h. After dehydrating graded ethanol, the critical point dried and sputtercoated with gold for 30 s. The diameter and arrangement of collagen fibers were viewed and recorded using SEM (SU8020, Hitachi, Japan). For TEM, excised tissues were fixed with 2.0% glutaraldehyde for 24 h and 2% osmium tetroxide for 2 h, then stained with lead citrate and uranyl acetate, and embedded in epoxy resin. An ultramicrotome (Leica, Germany) was used to prepare transverse and longitudinal sections. The ultrastructure of tendon tissues was observed and recorded by TEM (HT7700, Hitachi, Japan). The collagen fibrils and mitochondria from TEM images were analyzed individually in Image J software.

### Biomechanical Testing and Motor Function Analysis

The Achilles tendon was clamped in a jig, and the experiment was conducted on the universal testing systems (Instron5969, United States). The cross‐sectional area (mm^2^) of tendons was calculated by a Vernier caliper. The preload was 0.5 N, followed by 20 cycles of cyclic stretching with a load range of 0–15 N and a 10 mm min^−1^ loading rate. The Achilles tendon was constantly moistened with a saline needle during the test. After cyclic stretching, the Achilles tendon was stretched at a loading rate of 10 mm min^−1^. The mechanical properties of tendons were calculated by failure force (*N*), stiffness (N mm^−1^), and tensile modulus (MPa). The criterion for the experiment's success was that the Achilles tendon broke at the middle point but not at the fixing clamp position. To assess the motor function of injured tendons, their gait performance was evaluated by AFI, using a modified method as previously reported.^[^ ^62^ ^]^ Briefly, a defined roadway (about 90 cm in length and 20 cm in width) was filled with white paper. The hind paws were evenly covered with black ink then the rats could walk freely along the path and leave footprints on the paper. The related footprints parameters, including print length (PL), toe spreading length (the distance between the first and fifth toes, TS), and intermediary toe spreading length (the distance between the second and fourth toes, IT), was recorded and measured by Image J software. Next, based on the difference between the experimental (*E*) and the native (*N*) values, three related indicators, including print length factor (PLF), toe spreading length factor (TSF), intermediary toe spreading length factor (ITF), could be calculated by the established equations: PLF = (NPL− EPL)/EPL, TSF = (ETS − NTS)/NTS, ITF = (EIT − NIT)/NIT. Therefore, we could calculate the AFI values based on the equation below: AFI = 74 (PLF) + 161 (TSF) + 48 (ITF) − 5.^[^ ^64^ ^]^

### Statistical Analysis

GraphPad Prism 8 was used to conduct the statistical analysis. Data are shown as the mean ± standard deviation (SD). Multiple comparisons were performed by one‐way analysis of variance (ANOVA) with Tukey's post hoc test and Student's *t*‐test accomplished comparison between two groups. Statistical significance was set at *P* < 0.05. All experiments were independently performed at least three times.

## Conflict of Interest

The authors declare no conflict of interest.

## Author Contributions

S.W., Z.Y., and X.Z. contributed equally to this work. Y.O., Y.Q. and C.F. conceived the initial idea, designed the research, and revised the manuscript. S.W., Z.Y., and X.Z. performed the experiments, drafted the manuscript, and analyzed the data. J.L. and C.H. contributed to the data extraction and interpretation, image analysis, and figure production. All authors have read and approved the final manuscript.

## Supporting information

Supporting Information

## Data Availability

The data that support the findings of this study are available from the corresponding author upon reasonable request.
