# Supplementary material for: Energy‐Supporting Enzyme‐Mimic Nanoscaffold Facilitates Tendon Regeneration Based on a Mitochondrial Protection and Microenvironment Remodeling Strategy
Source: Adv Sci (Weinh). 2022 Aug 24;9(31):2202542. doi: 10.1002/advs.202202542 (PMC9631092; doi:10.1002/advs.202202542)
Supplement: Supplementary file 1 — Supporting Information [file ADVS-9-2202542-s001.pdf]

ADVANCED SCIENCE
Open Access

Supporting Information

for *Adv. Sci.*, DOI 10.1002/advs.202202542

Energy-Supporting Enzyme-Mimic Nanoscaffold Facilitates Tendon Regeneration Based on a Mitochondrial Protection and Microenvironment Remodeling Strategy

Shikun Wang, Zhixiao Yao, Xinyu Zhang, Juehong Li, Chen Huang, Yuanming Ouyang, Yun Qian* and Cunyi Fan**

Supporting Information

Energy-supporting Enzyme-mimic Nanoscaffold Facilitates Tendon Regeneration Based on a Mitochondrial Protection and Microenvironment Remodeling Strategy

Shikun Wang[1,2,3#], Zhixiao Yao[1,2,3#], Xinyu Zhang[4#], Juehong Li[1,2,3], Chen Huang[4], Yuanming Ouyang[1,2,3*], Yun Qian[1,2,3*], Cunyi Fan[1,2,3*]

1 Department of Orthopaedics, Shanghai Jiao Tong University Affiliated Sixth People’s Hospital, Shanghai, China.

2 Shanghai Engineering Research Center for Orthopaedic Material Innovation and Tissue Regeneration, Shanghai, China

3 Youth Science and Technology Innovation Studio, Shanghai Jiao Tong University School of Medicine, Shanghai, China

4 Engineering Research Center of Technical Textiles, Ministry of Education, College of Textiles, Donghua University, Shanghai, 201620, China

* CORRESPONDENCE

Yuanming Ouyang, PhD

E-mail: ouyangyuanming@163.com

Yun Qian, MD

E-mail: lollipopcloudland@foxmail.com

Cunyi Fan, PhD

E-mail: cyfan@sjtu.edu.cn

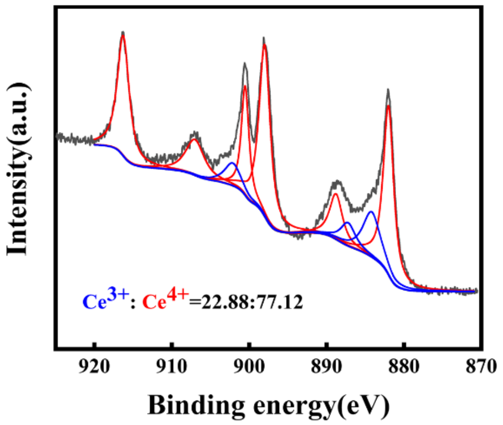

Figure S1 XPS spectrum of CeNPs. The XPS analysis of CeNPs indicated the oxidation state of Ce^{3+} and Ce^{4+} simultaneously coexisted at a ratio of 22.88:77.12.

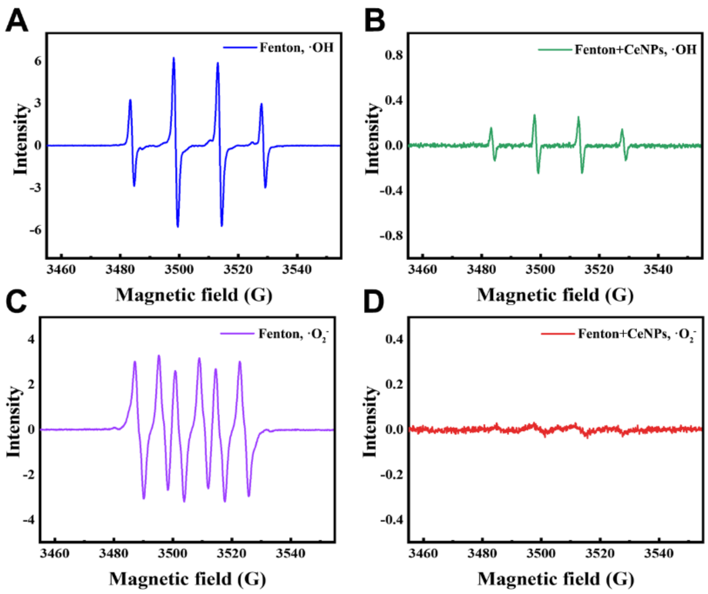

Figure S2 ESR spectra of different groups to examine the ROS scavenging properties of CeNPs. A ESR spectra of hydroxyl radicals generated by the Fenton reaction by using DMPO as the spin trapping agent at room temperature. B ESR spectra of hydroxyl radicals under the presence of Fenton agent and CeNPs. C ESR spectra of superoxide radicals generated by the Fenton reaction by using DMPO as the spin trapping agent at room temperature. D ESR spectra of superoxide radicals under the presence of Fenton agent and CeNPs.

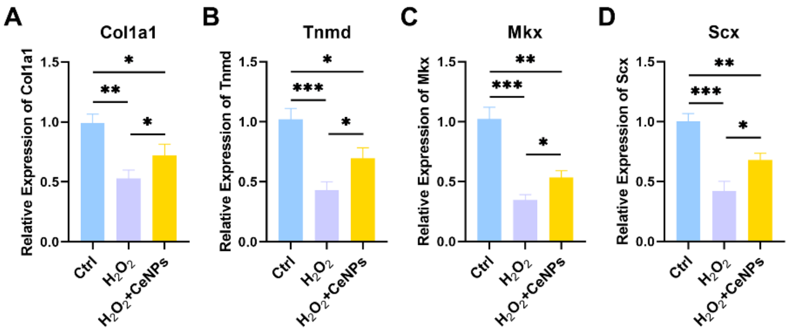

Figure S3 qRT-PCR of different groups to evaluate the mRNA expression level of tendon-specific markers in TDSCs. Semi-quantitative analysis of the relative expression level of Col1a1 (A), Tnmd (B), Mkx (C), and Scx (D) in TDSCs under different conditions in vitro (n=3 per group). Data are presented as mean ± SD. *P < 0.05; **P< 0.01; ***P< 0.001.
